# PKD2: An Important Membrane Protein in Organ Development

**DOI:** 10.3390/cells13201722

**Published:** 2024-10-17

**Authors:** Shuo Wang, Yunsi Kang, Haibo Xie

**Affiliations:** 1 MoE Key Laboratory of Evolution and Marine Biodiversity, Institute of Evolution and Marine Biodiversity, Ocean University of China, Qingdao 266003, China; wangshuo0782@gmail.com (S.W.); kangyunsi@ouc.edu.cn (Y.K.); 2 College of Marine Life Sciences, Ocean University of China, Qingdao 266003, China

**Keywords:** calcium, cilia, left–right asymmetry, organ development, PKD2, polycystic kidney disease

## Abstract

PKD2 was first identified as the pathogenic protein for autosomal dominant polycystic kidney disease (ADPKD) and is widely recognized as an ion channel. Subsequent studies have shown that PKD2 is widely expressed in various animal tissues and plays a crucial role in tissue and organ development. Additionally, PKD2 is conserved from single-celled organisms to vertebrates. Here, we provide an overview of recent advances in the function of PKD2 in key model animals, focusing on the establishment of left–right organ asymmetry, renal homeostasis, cardiovascular development, and signal transduction in reproduction and mating. We specifically focus on the roles of PKD2 in development and highlight future prospects for PKD2 research.

## 1. Introduction

PKD2 was identified by Mochizuki in 1996 from three family lineages of autosomal dominant polycystic kidney disease (ADPKD) patients [1]. Currently, approximately 1 in 400 to 1 in 1000 newborns are affected by ADPKD [2]. Mutations in PKD2 are estimated to account for approximately 15% of ADPKD cases [3]. In contrast, PKD1 mutations, responsible for around 85% of ADPKD cases, are associated with more severe disease outcomes, including larger kidney size, earlier onset of end-stage renal disease (ESRD), and more frequent complications like hypertension [4]. Despite this, PKD2 remains critical for understanding ADPKD due to its role in both renal and extrarenal tissues. PKD2 is expressed in the renal tubules of both *human* fetuses and adults, as well as in the epithelial of the liver, lung, bowel, brain, reproductive organs, and other tissues [5]. Additionally, PKD2 expression has also been observed in non-epithelial tissues, including vascular smooth muscle, skeletal muscle, myocardial cells, and neurons [5]. ADPKD is a multisystem disorder with lesions in other organs [6]. The interaction between the PKD1 and PKD2 gene products, polycystin-1 and polycys-tin-2, forms a complex involved in calcium signaling, which is crucial for tubular structure maintenance and cyst development. Therefore, elucidating the molecular mechanisms of organ development regulated by PKD2 is essential for the diagnosis and treatment of ADPKD.

Recent research has shown that PKD2 is a member of the TRP (transient receptor potential) ion channel family [7]. PKD2 contains six transmembrane domains: the first four TMHs (transmembrane helices) form a voltage-sensor-like domain, followed by a pore domain with two additional TMHs separated by a pore helix, a selectivity filter, and a second short pore helix [8]. The C-terminus of PKD2 contains EF-hand motifs that bind calcium ions, as well as a coiled-coil domain [9,10]. Cryo-electron microscopy shows that PKD2 assembles into homotetramers [8,11,12] and can also form heterotetramers with PKD1 [13]. PKD2 is typically localized in the endoplasmic reticulum (ER) and cilia. PKD2 forms non-selective calcium ion or voltage-activated cation channels in the ER [14,15]. Ciliary PKD2 is a non-selective cation channel protein involved in the transduction of mechanical signals [16,17,18,19,20]. While significant progress has been made in understanding the cellular localization and structure of PKD2, more attention is now being paid to elucidating its physiological functions in complex organisms.

**Figure 1:**
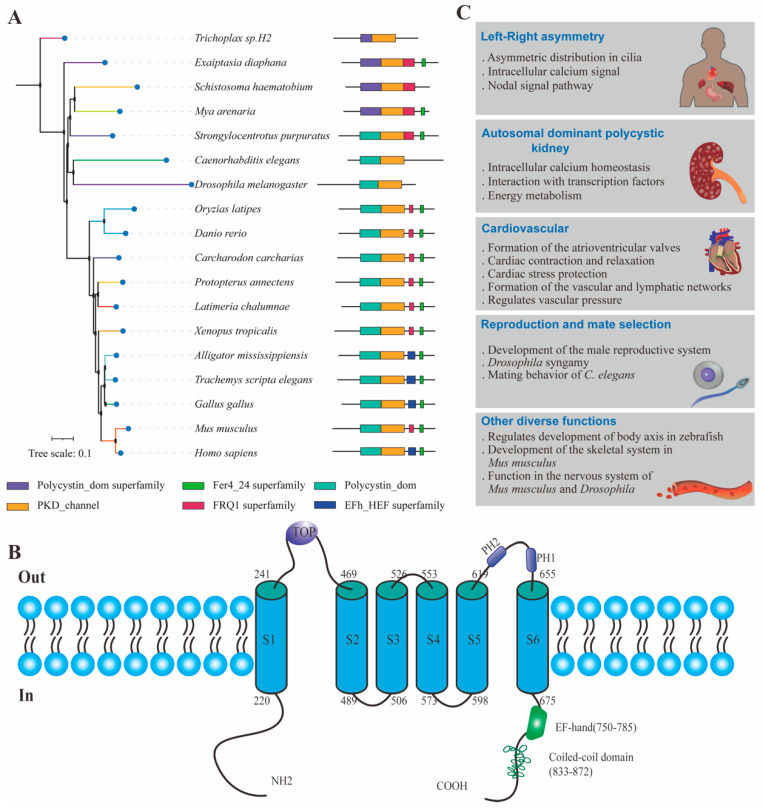
PKD2 is conserved across diverse species and participates in organ development. (**A**) Phylogenetic tree of PKD2 across diverse species. PKD2 homologs were obtained from NCBI. The Accession Numbers are RDD46437, XP_030835833, XP_020911675, NP_609561, XP_052762183, XP_051064762, NP_502838, XP_043917296, XP_006007388, XP_041048068, NP_001002310, NP_001129990, XP_004911138, XP_034627128, XP_019343674, XP_063165668, NP_001026311, NP_000288, NP_001178863, NP_032887 and XP_028704444. The neighbor—joining phylogenetic trees were constructed using MEGA X with default parameters and bootstrapping with 10,000 replications was conducted to evaluate the phylogenetic tree. The conserved domain was annotated using CDD Tools. (**B**) PKD2 is involved in establishing left–right organ asymmetry, maintaining renal homeostasis, cardiovascular development, and signal transduction in reproduction and mating. (**C**) Schematic diagram of the *human* PKD2 structure.

PKD2 is conserved in most animals (Figure 1A) [21,22,23,24,25,26,27,28,29,30,31]. Recent research shows diverse functions of PKD2 in various organisms, especially in multicellular ones [32,33,34,35,36,37,38,39]. Therefore, elucidating the diverse physiological functions of PKD2 using various model organisms, characterizing its functional properties in specific organs, and deciphering the common mechanisms underlying developmental abnormalities caused by PKD2 dysfunction are focal points in developmental biology. Here, we highlight the research progress on PKD2 in model species from the perspective of organ development (Figure 1B) and discuss future research directions for understanding PKD2’s physiological functions.

## 2. PKD2 and Left–Right Asymmetry

Internal organs are asymmetrically distributed in most vertebrates. In *humans*, incorrect left–right asymmetry of internal organs is often associated with disorders, such as congenital heart disease [40,41]. PKD2 plays an essential role in establishing left–right asymmetry during the embryonic development of vertebrates [42]. PKD2 dysfunction results in defective left–right patterning in *humans*, *mice*, *zebrafish*, and *frogs* [32,33,34,35].

**Figure 2:**
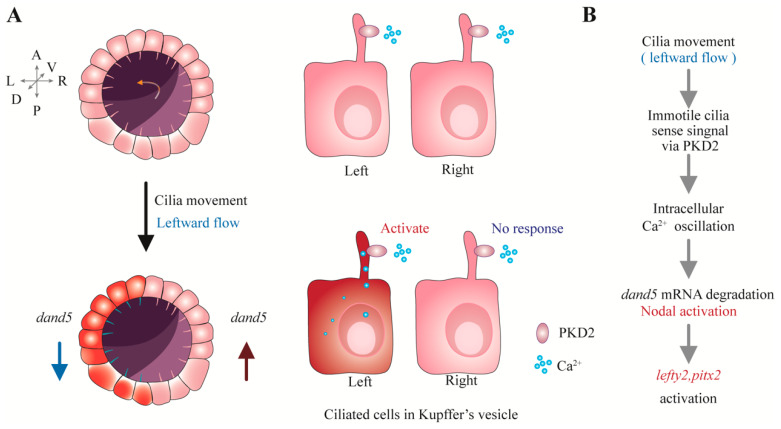
PKD2 is involved in establishing left–right asymmetry. (**A**) Mechanism of PKD2 in left–right asymmetry of *zebrafish*. At the bud stage, immotile cilia in the left–right organizer (Kupffer’s vesicle, KV) maintain bilateral symmetry (top panel). At the 1 to 4 somite stage, motile cilia in KV generate a leftward flow (orange), which triggers the PKD2-dependent ciliary calcium oscillation on the left side of KV (bottom panel). Calcium oscillation inhibits dand5 expression and activates Nodal signaling in left-side cells, which further induces expression of Lefty2 and Pitx2 in the left side of KV. (**B**) General molecular mechanism of PKD2 in establishing left–right asymmetry. Motile cilia in the LRO (left–right organizer) generate leftward fluid flow. Immotile cilia detect mechanical signals via PKD2 and convert them into intracellular calcium signals. Intracellular calcium signals inhibit dand5 expression, which activates Nodal signaling. Consequently, Lefty2 and Pitx2 are expressed on the left side of LRO, promoting molecular processes of organ development in left–right asymmetric pattern.

The left–right organizer (LRO) is a key tissue for left–right patterning, including the node in *humans* and *mice* [33], Kupffer’s vesicle in *zebrafish* [34], and the gastrocoel roof plate in *frogs* [43,44]. Primary and motile cilia are abundant in the LRO, where the coordinated beating of motile cilia generates fluid flow towards the left side, allowing immotile cilia to sense the mechanical signals from the leftward flow and initiate the first asymmetric event in embryonic development [45,46,47]. Mechanical force causes the primary cilia on the left side of the LRO to bend towards the ventral side and the primary cilia on the right side to bend towards the dorsal side, resulting in greater tension on the dorsal side of the left cilia, which activates PKD2 enriched on the dorsal side and induces changes in calcium signaling in both the cilia and cytoplasm of the left-side cells of the LRO (Figure 2A) [19,20,46]. In *zebrafish*, the stronger bending of immotile cilia on the left side of the KV is the reason for the calcium signals in the left-side cells of the LRO [19]. Defects in the motility of cilia in the LRO lead to abnormal left–right asymmetry in organ development [44]. Left–right asymmetry defects in *zebrafish c21orf59* (motile cilia gene) morphants can be counteracted by using optical tweezers to manipulate the primary cilia on the left side of Kupffer’s vesicle, but this method cannot rescue the left–right asymmetry defects in *zebrafish pkd2* morphants [19]. Consistent with *zebrafish*, mechanical stimuli administered to primary cilia by optical tweezers also trigger signals for the establishment of left–right patterning in *mice* [19]. These data confirm that PKD2 acts as a mechanosensor on the ciliary membrane to regulate calcium signaling in the left-side cells of LRO, directly promoting the establishment of left–right asymmetry of internal organs [19,20].

Activation of calcium signaling mediated by PKD2 in left-side cells of the LRO leads to Bicc1 interacting with Ccr4 to form a degradation complex for *Dand5* mRNA, ultimately inhibiting Dand5 expression in the left-sided lateral plate mesoderm [48,49,50]. Degradation of Dand5 activates Nodal signaling in the left lateral plate mesoderm and enhanced Nodal signaling activates the expression of Lefty2 and Pitx2 in the left lateral plate mesoderm (Figure 2B) [49,51,52,53,54,55,56,57,58]. These events collectively determine the establishment of left–right asymmetry in internal organs [59].

## 3. PKD2 and Kidney

PKD2 plays a crucial role in maintaining the structure and function of the kidney. Mutations in PKD2 can lead to autosomal dominant polycystic kidney disease (ADPKD), a multisystem and progressive adult-onset genetic disorder characterized by the formation of fluid-filled cysts and increased kidney volume [60]. The “two-hit” model has been proposed to explain the genetic mechanism of renal cyst formation caused by PKD2 mutations [61,62]: (1) Both proximal and distal tubular epithelial cells of all nephrons carry both mutant and non-mutant PKD2 alleles, which means the individual has a heterozygous germline mutation, which represents the first hit; (2) injuries or other factors may cause mutations in the non-mutant PKD2 allele, resulting in somatic mutations, which represents the second hit, leading to insufficient PKD2 levels in the cells.

### 3.1. Function of PKD2 in Mouse Kidney

Both *mouse* and *human* kidneys belong to the metanephros, indicating that they share a structure that is highly similar in nature [63,64]. Pkd2 mRNA expression is detectable in the cortical region of adult *mouse* kidneys [65,66]. PKD2 is also expressed in the loop of Henle and distal convoluted tubules of *mouse* kidneys, where it localizes to the basal lateral membrane and cilia of epithelial cells [67,68]. Homozygous mutants of *mouse* Pkd2 die at embryonic day 13.5, exhibiting defects in the atrioventricular septum, renal cysts, and pancreatic cysts, while heterozygotes show milder pancreatic cysts, suggesting a dosage-dependent regulation of tissue function in *mouse* kidneys by PKD2 [69].

#### 3.1.1. PKD2 Regulates Intracellular Calcium Homeostasis in Renal Cells

In *mouse* kidneys, PKD2 on cilia responds to mechanical signals from extracellular fluid flow, triggering changes in intracellular calcium signaling. Ryanodine receptors are located on the endoplasmic reticulum and are involved in the release of calcium ions from intracellular calcium stores into the cytoplasm [70]. Ryanodine receptor inhibitors can block this signal transduction, highlighting the significance of Ryanodine receptors in this process [68]. PKD2 in the ER interacts with the N-terminus of inositol 1,4,5-trisphosphate receptor (IP3R) via its C-terminus, a process activated by low calcium that leads to the release of higher calcium from the ER [71]. PKD2 shows higher permeability to potassium ions than to calcium ions. Overexpression of the trimeric intracellular cation channel TRIC-B (potassium channel) corrects defects in ER calcium ion release and inhibits cyst formation, whereas TRIC-B deficiency exacerbates cyst formation in Pkd2 heterozygous kidneys, which suggests that PKD2 in the ER may function as a potassium ion channel, facilitating potassium–calcium ion exchange and thereby regulating intracellular calcium ion concentrations through IP3R-mediated mechanisms [72]. PKD2 is localized to the cell membrane of *mouse* inner medullary collecting duct cells and Madin–Darby canine kidney cells, functioning as an ion channel, which suggests that external signals can be transduced by PKD2 on the cell membrane [73]. Post-translational modifications of PKD2, specifically phosphorylation of Ser812 by Casein Kinase II, play a role in regulating how PKD2 functions as an ion channel [74]. Single-channel studies show that both PKD2 and its S812A variant function as divalent cation channels with comparable current amplitudes under various holding potentials, exhibiting open probabilities dependent on cytoplasmic calcium ions in a bell-shaped manner; however, S812A shows significantly reduced sensitivity to calcium ion activation/inactivation, being 10-fold less sensitive than the wild-type PKD2 channel [74].

#### 3.1.2. PKD2 Regulates Cell Proliferation in *Mouse* Kidney

**Figure 3:**
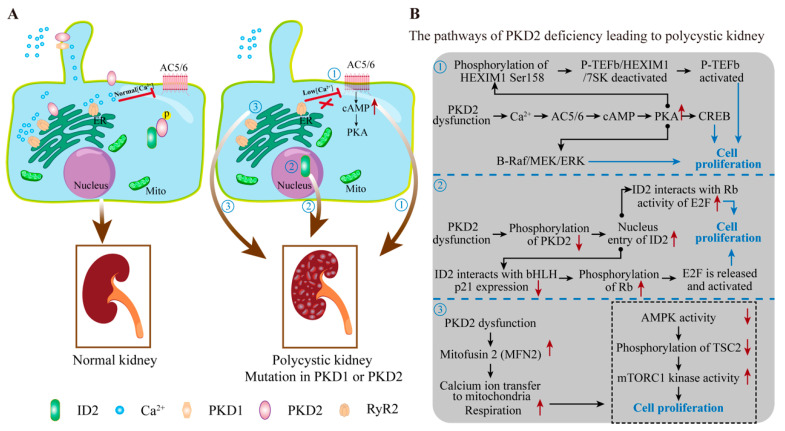
Dysfunction of PKD2 results in polycystic kidney. (**A**) PKD2 plays a key role in maintaining intracellular calcium homeostasis under normal physiological conditions (left). PKD2 dysfunction disrupts this balance, leading to polycystic kidney disease (right). (**B**) Three key mechanisms are involved in polycystic kidney development due to PKD2 dysfunction: (1) PKD2 dysfunction reduces intracellular calcium, increasing cAMP levels, which enhances protein kinase A (PKA) activity [77,78,79,80,81]. PKA promotes cell proliferation via HEXIM1 inactivation and CREB/B-Raf/MEK/ERK signaling pathways [82,83,84,85]. (2) Reduced PKD2 phosphorylation causes ID2 retention in the nucleus, leading to E2F transcription factor activation and subsequent cell proliferation. ID2 also represses P21 expression, further enhancing E2F release [86,87,88]. (3) Increased mitofusin 2 (MFN2) expression enhances mitochondrial calcium uptake and respiration, promoting cell proliferation [89]. Studies on PKD1 indicate increased respiration inhibits AMPK, reduces TSC2 phosphorylation, and activates mTORC1, further driving cell proliferation [90,91].

Over-proliferation of renal tubular epithelial cells occurs in both heterozygous and homozygous Pkd2 mutant *mice* before cyst formation [75]. Furthermore, reports indicate that PKD2 is overexpressed in *human* renal cyst tissues and can induce renal cysts in *mice* when overexpressed under model *human* PKD2 conditions [5,76]. The causes of polycystic kidney disease due to PKD2 mutation can currently be categorized into three main aspects (Figure 3A,B).

#### 3.1.3. PKD2 Regulates Cell Proliferation through Calcium Signaling

In the cystic epithelial cells of Pkd2 mutant *mouse* kidneys, decreased intracellular calcium ions reduce the calcium-mediated inhibition of adenylyl cyclase 5/6 (a plasma membrane-bound enzyme with multiple calcium-sensitive subtypes), thereby increasing cellular cAMP levels [77,78,79,80]. Elevated cAMP levels in the cholangiocytes resulting from PKD2 deficiency enhance protein kinase A (PKA) activity, which leads to ERK phosphorylation (pERK), stimulating VEGF signaling, which ultimately activates cell proliferation and promotes the formation of hepatic cysts [81]. In the kidneys, activated PKA can phosphorylate cAMP responsive element-binding protein (CREB), driving the transcription of target genes in pathways related to cell proliferation [82]. Recent studies show that PKA phosphorylates serine158 of HEXIM1, which inactivates the P-TEFb/HEXIM1/7SK snRNP complex, releasing and activating the central transcriptional elongation regulator (positive transcription elongation factor b, P-TEFb), thereby promoting cell proliferation [83]. Moreover, P-TEFb is overexpressed in *mouse* and *human* renal cysts, and the activation of P-TEFb facilitates the establishment of the *zebrafish* renal cyst model [83]. Overexpression of *human* PKD2 in *mice* results in mitotic instability and centrosome overduplication of renal cells and promotes cell proliferation, which may be linked to excessive activation of the B-Raf/MEK/ERK phosphorylation by PKA (Figure 3B) [84,85].

#### 3.1.4. PKD2 Interacts with Transcription Factors to Regulate Cell Proliferation

Phosphorylated PKD2 (S812) interacting with the inhibitor of DNA Binding 2 (ID2) sequesters the ID2-E47 complex in the cytoplasm and prevents ID2 from inhibiting p21 activation, which reduces cyclin-dependent kinase 2 (CDK2) activity and inhibits cell proliferation [86,87]. Enhanced nuclear localization of ID2 has been observed in *human* and *mouse* renal cysts, suggesting that ID2 plays a pivotal role in PKD2-mediated cell cycle regulation [86,87]. Mutations in PKD2 result in increased nuclear ID2 levels, potentially regulating the activation of E2F transcription factors by interacting with the retinoblastoma (Rb) transcriptional corepressor, thereby enhancing cell proliferation [88]. Elevated ID2 levels may also bind basic helix–loop–helix transcription factors (bHLH) to suppress p21 gene expression, which enhances CDK2/CDK4-mediated Rb phosphorylation, resulting in the release of E2F from the Rb-E2F complex and cell proliferation (Figure 3B) [88].

#### 3.1.5. PKD2 Regulates Cell Proliferation through Energy Metabolism

Mitofusin 2 (MFN2, an outer mitochondrial membrane GTPase facilitating mitochondria-ER proximity) expression was increased in PKD2 knockdown cell culture models and cyst-lining epithelial cells from *human* ADPKD kidneys, which enhances calcium transfer efficiency into mitochondria and promotes mitochondrial respiration (Figure 3B) [89]. Knockdown of MFN2 restored defective mitochondrial calcium ion transfer and reduced cell proliferation in *mouse* with kidney cysts, suggesting that PKD2 acts as a regulator of mitochondrial biogenesis and bioenergetics by regulating essential mitochondrial proteins at the ER–mitochondrial junction [89]. Studies on PKD1 indicate that increased proliferation of cystic renal epithelial cells elevates intracellular ATP levels, which inhibit AMP-activated protein kinase (AMPK) activity and prevents AMPK from phosphorylating tuberous sclerosis complex 2 (phosphorylated TSC2 suppresses mTORC1 kinase activity), leading to enhanced protein synthesis and cell proliferation (Figure 3B) [90,91].

#### 3.1.6. PKD2 Maintains the Polarity of Cells

Epithelial cell polarity is crucial for maintaining the structural and functional integrity of the kidney. *Pkd2* mutant *mice* exhibit increased expression of β-catenin, axin2, and cMyc, indicating disrupted canonical Wnt signaling [92]. Disrupted E-cadherin-mediated cell–cell contacts result in significantly elevated cytoplasmic β-catenin levels, which impede renal epithelial polarization [92].

#### 3.1.7. PKD2 Is Involved in the Development of Cilia in *Mouse* Kidneys

In vitro experiments demonstrate that PKD2-mediated mechanotransduction regulates cilia length in mammalian epithelial and mesenchymal cells [93]. However, the regulation of ciliary length by PKD2 remains controversial. There are fewer and shorter cilia in renal epithelial cells of PKD2-deficient *mice* [92], while the cilia of PKD2-deficient cyst-lining tubule cells are longer than the control group from another report [17]. Interestingly, the study showed that cilia integrity contributes to renal cyst formation caused by PKD2 deficiency, although the mechanism of this is not clear [94]. Recent studies have shown that the loss of PKD1 in *mice* alters the biomechanical properties of the basement membrane of renal tubules in a cilia-dependent manner, ultimately resulting in distal renal tubule dilation and cyst formation independent of cell proliferation [95]. This discovery may suggest a potential similar regulatory role for PKD2 in cystogenesis.

### 3.2. Function of PKD2 in Zebrafish Kidney

*Zebrafish* have pronephros during the embryo stage and mesonephros during the adult stage, with nephron units segmented similarly to mammalian kidneys [63]. Pkd2 in *zebrafish* is located at the basal lateral membrane of the pronephric duct [96]. *Zebrafish pkd2* mutants do not develop polycystic kidneys [97]. However, there are pronephric cysts in the *pkd2* morphant due to pronephric duct blockage [37,96,98]. Knockdown of Pkd2 reduces levels of Scribble at the basal membrane, inhibiting the Hippo signaling pathway and causing cytoplasmic YAP to translocate to the nucleus, which promotes glomerular cyst formation [99]. The N-terminal GSK-3β phosphorylation site of Pkd2 facilitates its localization to the lateral cell membrane [100], while the C-terminal acidic cluster/PACS binding domain promotes Pkd2 retention in the ER/Golgi membrane [96,101]. Mislocalization of Pkd2 affects the structural integrity and function of *zebrafish* renal tissues [96,100]. However, current research suggests that Pkd2 does not directly participate in the physiological function of *zebrafish* kidneys through cilia development and function [96,98].

### 3.3. Progress in Clinical Drug Research on ADPKD

Recent advancements in understanding the pathophysiology of diseases have led to new potential therapeutic targets. PKD2 regulates cell proliferation through calcium signaling. For example, Tolvaptan, a vasopressin V2 receptor antagonist, reduces cAMP levels and is currently used to slow the progression of Autosomal Dominant Polycystic Kidney Disease (ADPKD) [102]. Additionally, somatostatin analogs, such as Octreotide and Lanreotide, have been reported to delay the growth of renal cysts [103]. The pan-histone deacetylase (HDAC) inhibitor, trichostatin A, has also shown promise in reducing cyst progression and slowing kidney function decline [88]. Moreover, recent studies indicate that everolimus and sirolimus, both mTOR inhibitors, can slow renal cyst progression [104]. PKD2 maintains cell polarity, and while promising β-catenin inhibitors are under investigation, their approval for ADPKD remains uncertain [105].

## 4. PKD2 and Cardiovascular

### 4.1. PKD2 Regulates Cardiac Development

#### 4.1.1. PKD2 Is Involved in the Formation of the Atrioventricular Valves

In *zebrafish*, early blood flow in the heart generates mechanical signals. Pkd2 of atrioventricular endocardial cells transduces mechanical signals to regulate intracellular calcium ion concentration, and then calcium ion/calmodulin-dependent protein kinase Camk2g activates the expression of *klf2a* to promote valve formation [38,106,107].

#### 4.1.2. PKD2 Is Involved in Cardiac Contraction and Relaxation

In *mouse* cardiomyocytes, PKD2 can interact with the cardiac ryanodine receptor (RyR2) [108]. The C-terminus of PKD2 functionally inhibits RyR2 channel activity in the presence of calcium, resulting in altered calcium signaling in the heart [108]. Additionally, heterozygous PKD2 decreases calcium ion concentration in the sarcoplasmic reticulum, which leads to increased PKA-mediated phosphorylation of cardiac troponin I and decreased β-adrenergic signaling-mediated dephosphorylation of phospholamban (PLB) and ultimately altering myocardial contractility [109]. Similarly, *pkd2* mutation in *zebrafish* dysregulates calcium ion homeostasis in cardiomyocytes, leading to abnormal cardiac contraction and relaxation [110]. PKD2 is also expressed in intestinal smooth muscles in fruit flies and PKD2 deficiency reduces food intake and waste excretion in larvae, which indicates that PKD2 regulates the contractility of smooth muscle [39]. Additionally, PKD2 regulates the contractility of tracheal smooth muscle in *mice* [111].

#### 4.1.3. PKD2 Is Involved in Cardiac Protection under Stress

Under stress, *mice* exhibited increased left ventricular mass along with elevated levels of PKD2, chromogranin B (CGB, an upstream regulator of BNP), and natriuretic peptides (ANP and BNP) [112]. After stress cessation, the left ventricular mass returned to baseline [112]. However, PKD2-deficient *mice* failed to upregulate cardioprotective atrial natriuretic peptides during stress and their left ventricular mass failed to recover after stress cessation, suggesting the involvement of PKD2 in cardiac protection during emergencies [112].

### 4.2. PKD2 Is Involved in Vascular Development

PKD2 localizes at the sarcoplasmic reticulum and ciliary membrane in the smooth muscle cells of *mouse* aortas [113,114,115]. Abnormal function of PKD2 is linked to hypertension and structural vascular abnormalities, including intracranial aneurysms [116]. Hypertension associated with PKD2 deficiency can manifest in childhood and precede the onset of kidney disease [117].

#### 4.2.1. PKD2 Is Involved in the Establishment of Vascular and Lymphatic Networks 

In *mice*, homozygous *Pkd2* mutants display severe edematous vascular phenotypes linked to lymphatic developmental defects, including reduced lymphatic vessel density, impaired vascular branching, and abnormal migration of early lymphatic endothelial cell precursors [118]. Knockdown of *pkd2* in *zebrafish* results in abnormal development of the caudal vein [119]. Live imaging shows that endothelial cilia bend in response to blood flow in *zebrafish* embryos and affect intracellular calcium concentrations, which indicates that Pkd2, located in cilia, senses blood flow shear forces to regulate vascular network development [119]. Moreover, *Zebrafish* Pkd2 senses radial shear force and circumferential pressure from blood flow, participating in vascular morphogenesis through the regulation of endothelial cell rearrangement, shape changes, cell division orientation, and extrusion/layering [120]. Although PKD2 typically functions alongside primary cilia, suggesting that its role may depend on the presence of cilia, studies indicate that the regulation of endothelial cell area appears to be achieved through cilia-mediated flow sensing. In contrast, the regulation of endothelial cell extrusion may rely on cilia-independent mechanosensitivity mediated by PKD2 [120]. This finding reveals distinct regulatory mechanisms.

#### 4.2.2. PKD2 Regulates Vascular Pressure by Sensing Mechanical Forces Generated by Blood Flow

Research shows that PKD2 located in the cilia of vascular endothelial cells in both *mice* and *humans* senses fluid shear stress through a complex biochemical pathway involving calcium, calmodulin, protein kinase B, and protein kinase C [115]. When exposed to fluid shear stress, *mouse* endothelial cells can produce nitric oxide to regulate blood pressure [115]. Studies have shown that blood flow activates PKD2 channels in endothelial cells, which causes calcium influx, potassium channel activation, phosphorylation of endothelial nitric oxide synthase, vascular hyperpolarization, dilation, and ultimately reduces blood pressure [121,122]. Intravascular pressure and α1-adrenoceptors activate PKD2 channels in arterial myocytes to regulate blood pressure by inducing an inward sodium current, membrane depolarization, and vasoconstriction [123]. Furthermore, vascular pressure and the SUMOylation dynamics of PKD2 stabilize vascular pressure by controlling PKD2 abundance on the cell membrane [124]. For example, high intravascular pressure promotes SUMO-PKD2 localization to cell membranes and low intravascular pressure results in the degradation of SUMO-PKD2 in lysosomes. Intravascular pressure activates SUMO-PKD2, not PKD2, channels, through this SUMOylation-dependent mechanism and intravascular pressure regulates the surface density of SUMO-PKD2-mediated Na^+^ currents (INa) in myocytes to control arterial contractility [124]. In heterozygous Pkd2 *mice*, mesenteric vessels exhibit impaired relaxation function, primarily due to increased vascular oxidative stress, which reduces NO bioavailability [117].

## 5. PKD2 and Reproduction and Mating

Defects in PKD2 function frequently result in male infertility and reproductive defects in *humans* [125,126]. PKD2 plays a role in the reproductive system of male *mice* via the TGF-β/BMP and canonical Wnt signaling pathways [127]. Disruption of PKD2 results in mesonephric tubule/efferent duct dilation, epididymal coiling failure, and defective testicular development [127]. PKD2 is also expressed in the testes of *Drosophila* and in all stages of sperm precursor differentiation, except for germline stem cells [128]. In mature sperm, PKD2 is found in punctate patterns at the acrosome, head, and tail [129]. Sperm with PKD2 defects have intact structures but with reduced motility [128,129,130,131,132].

In *C. elegans*, PKD-2 (the homolog of *human* PKD2) plays a role in mating behavior. PKD-2 is expressed in CEphalic Male Sensory Neuron (CEM) of the head, and Ray neuron B (Ray B), and HOok Neuron B (HOB) of the tail [133,134]. Neurons expressing PKD-2 feature an exposed cilium at the distal end of the dendrite [135]. PKD-2 localizes to the endoplasmic reticulum (ER), dendritic puncta, the cilium, and extracellular vesicles (ECVs) [133,136]. ECVs are essential for chemotaxis towards mates, response to mate contact, and locating the mate’s vulva [133,137,138,139]. ECVs containing PKD-2 are released from the tip of the nose (where CEM cilia are exposed) and male tail rays (where RnB and HOB cilia are exposed) [140]. These ECVs are directionally transferred to the hermaphrodite’s vulva during *C. elegans* mating [141]. ECVs containing PKD-2 induce male tail-chasing behavior, where the tail curls to contact its own head and moves in a backward circle [140]. Tail curling enables the male to contact and circle around the hermaphrodite body during mating and the release ratio of ECVs containing PKD2 increases during male mating [140,142]. Transcriptome sequencing of ECVs revealed new pathways that control ECV biogenesis, polycystin signaling, and ECV cargo [143]. For example, the proteins that participate in polycystin signaling pathways, including homologs of Tumor Necrosis Factor (TRAF), *trf-1*, *trf-2*, and the p38 MAPK *pmk-1*, influence male mating behaviors [143]. Deficiency and mislocalization of PKD-2 both impact mating behavior [135,136,144,145,146,147,148,149,150,151,152,153].

## 6. Other Diverse Functions of PKD2

### 6.1. Pkd2 Regulates the Development of Body Axis in Zebrafish

Loss of Pkd2 function in *zebrafish* results in dorsal curvature of the body axis [37]. Preliminary research indicates that Pkd2 maintains cellular calcium ion homeostasis and participates in negative feedback control of embryonic collagen gene expression via the phosphoinositide 3-kinase signaling pathway [154]. Loss of Pkd2 function upregulates Sec23A, Sec23B, Sec24C, and Sec24D, resulting in excessive accumulation of type II collagen in notochord sheath cells that may be responsible for dorsal curvature of the body axis in *zebrafish* [154,155]. Overexpression of Pde1a in *zebrafish pkd2* morphants reduces cAMP concentration and mitigates axial curvature, which suggests that Pkd2 may influence axial morphology through the modulation of cAMP levels [156]. Drug screening shows that inhibitors of transforming growth factor receptor ALK5 kinase, diclofenac sodium, pyrithione zinc, 2,5-di-t-butyl-4-hydroxyanisole, and histone deacetylase (TSA) can inhibit tail curvature caused by Pkd2 deficiency [157,158].

### 6.2. PKD2 Is Involved in the Development of Skeletal System in Mice

Studies indicate that Pkd2 likely plays a crucial role in craniofacial growth. *Mice* with conditional deletion of *Pkd2* in neural crest-derived cells exhibited signs of mechanical trauma, including fractured molar roots, distorted incisors, alveolar bone loss, compressed temporomandibular joints, and abnormal skull shapes [159]. In addition, *mice* with conditional deletion of *Pkd2* in mature osteoblasts showed reduced bone mineral density, trabecular bone volume, cortical thickness, mineral apposition rate, and impaired biomechanical properties of bone [160]. Moreover, Pkd2 deficiency in mature osteoblasts also leads to decreased expression of peroxisome proliferator-activated receptor γ (PPARγ), reduced bone marrow fat and adipogenesis [160]. Current research suggests that PKD2 regulates the generation of the adipocytes and osteoblasts by modulating PPARγ and Runx2 [160].

### 6.3. Function of PKD2 in Nervous System

PKD2 in Class III (CIII) multidendritic sensory neurons of *Drosophila* is involved in sensing noxious cold [161]. Collar-receptor neurons in Platynereis larvae express PKD2-1 (the homolog of *human* PKD2) to detect environmental water flow, which may trigger a coordinated whole-body response to avoid predators [162]. In *mice*, PKD2 is localized to the cilia of ependymal cells, where it responds to shear force from cerebrospinal fluid [163]. The process contributes to the polarization of ependymal and choroid plexus cells by regulating the asymmetric localization of VANGL2 on the apical surface of choroid plexus cells and ciliary position [163]. Additionally, PKD2 balances proliferation and differentiation of neural progenitor cells through the Notch signaling pathway and the STAT3 transcription factor in *mice* [164].

## 7. Perspectives

ADPKD is a prevalent autosomal dominant genetic disorder, affecting 1 in 500–2500 individuals worldwide [6,165]. ADPKD is a multisystem disorder affecting the kidneys, liver, pancreas, arachnoid membrane, and heart [3,166,167,168], with 15% of patients harboring PKD2 mutations [3]. Therefore, studying PKD2 in model organisms helps elucidate its pathogenic mechanisms. Researchers have explored PKD2 functions in establishing left–right asymmetry, renal function maintenance, cardiovascular development, and signal transduction of reproductive and mating, including *mice*, *zebrafish*, *C. elegans*, *Drosophila*, and *Xenopus laevis* (Table 1). These studies provide crucial data for diagnosing, treating, and developing drugs for related diseases.

However, technological limitations hinder a comprehensive understanding of PKD2 function, and specific mechanisms in certain biological processes require further elucidation. The study of PKD2 using *zebrafish* has faced challenges due to the lack of highly specific antibodies, hindering the analysis of PKD2’s tissue distribution and subcellular localization, and limiting a comprehensive investigation of its role in organ development. Additionally, direct observation of PKD2’s polar localization in cilia in vivo could help provide insights into its role in left–right asymmetry. Although preliminary conclusions have been drawn in *mice*, the imaging resolution is low, and these findings have not been validated in other animal models [19,20]. Therefore, developing high-resolution imaging techniques represents a promising direction for future research. In left–right asymmetry, future research could explore how PKD2’s polarity localization in cilia is determined, the function of PKD2 in LRO centrosome polarization, and the role of calcium ions in Dand5 mRNA degradation. Left–right asymmetrical defects can lead to organ displacement and affect organ function, such as congenital heart disease [169]. PKD2 is currently thought to act as a mechanical sensor in regulating the asymmetric development of the left and right organs, and its mutation can lead to congenital heart disease [170]. Therefore, the development of related molecular drugs to treat congenital heart disease induced by PKD2 dysfunction is also an important direction in the future.

Recent studies indicate that intact cilia promote cyst formation in PKD2-mutant kidneys, challenging the current theory that PKD2 functions through cilia in the kidneys [94]. The discovery that reintroducing the TRIC-B potassium channel can rescue kidney cysts offers a new perspective for identifying drug targets to treat PKD2-induced polycystic kidney disease [72]. Some suggest that PKD1 deletion in *mice* alters the biomechanical properties of the renal tubular basement membrane in a cilia-dependent manner, leading to distal tubular dilation and cyst formation, independent of cell proliferation [95]. Whether PKD2 is also involved in similar physiological functions needs to be further explored.

Besides embryonic left–right asymmetry and autosomal dominant polycystic kidney disease, PKD2 is also involved in the development of other organs in model organisms. Corresponding diseases occur in *humans*, but underlying mechanisms are unclear. Further research is needed to understand how PKD2 contributes to cardiac development, vascular network formation, and vascular constriction. The roles of PKD2 in mammalian reproductive development, regulation of sperm motility in Drosophila, and mating of *C. elegans* are not fully understood. The mechanism by which loss of Pkd2 function causes body axis curvature in *zebrafish* also requires further investigation. One potential avenue for future research is to explore whether PKD2 influences body axis morphology either directly or indirectly by modulating neural activity and muscle contraction. Diverse subcellular localization of PKD2 contributes to its functional diversity. Therefore, employing high-resolution microscopy to study subcellular localization of PKD2 in diverse organs is crucial for understanding its function.

The current tools used to study PKD2 include the *zebrafish* mutant. The *cup* and *hi4166* mutant serve as a significant model for studying PKD2 function in *zebrafish* [97,157]. For *mouse* models, the *mouse* mutant is used, including Pkd2^nf3/nf3^ and Pkd2^WS25/−^, which provides essential insights into the role of PKD2 in kidney development and function [171,172]. For overexpression models, the PKD2-Y transgenic *mouse* has been used to investigate the effects of PKD2 overexpression on kidney physiology [76]. In addition, the PKD2 mutant *amo* in *Drosophila* offers a unique perspective on PKD2 biology in invertebrate models [128]. The PKD-2 mutant in *C. elegans* has also been utilized to elucidate PKD2-related functions, particularly in the context of developmental processes [133]. Future priorities for the development of PKD2 animal models may include *zebrafish* gene knock-in. Employing *zebrafish* gene knock-in techniques to trace the expression patterns and subcellular localization of PKD2 could enhance our understanding of its biological roles. Utilizing conditional knockout models in both *zebrafish* and *mice* could also help determine whether PKD2 plays a critical role in kidney development and function during different life stages. Integrating these tools will not only deepen our understanding of PKD2’s functions but also advance future research in related fields.

Different animal models present distinct advantages and limitations for studying PKD2 function. (1) *Mouse* models offer another valuable approach, with various PKD2 knockout and transgenic strains providing insights into the gene’s role in kidney development and disease progression. These models closely mimic ADPKD [173]. However, genetic and environmental variations between species can complicate the translation of findings to clinical settings [173]. (2) *Zebrafish* models are particularly advantageous due to their transparency during early development and the ease of gene manipulation. This allows for real-time observation of kidney development and cyst formation. However, the physiological differences between *zebrafish* and mammals may limit the direct applicability of findings to *human* conditions [174,175]. (3) *Drosophila* and *C. elegans* models are simpler organisms that can be useful for genetic studies and high-throughput screening of potential therapeutic compounds. Nonetheless, their simplified biological systems may not fully capture the complexities associated with mammalian kidney disease [176]. In summary, while each animal model has unique strengths that can contribute to our understanding of PKD2 and its implications for *human* health, the limitations inherent in each model must also be carefully considered when interpreting results.

Besides model organisms, investigating the pathogenic mechanisms and functions of PKD2 using clinical samples and organoids is gaining traction. *Human* kidney organoids are promising for drug screening. Currently, collecting ducts cannot form in kidney organoids. Compared to in vivo conditions, kidney organoids have smaller cyst volumes and lack urine flow and vasculature [177,178,179]. Thus, improving culture conditions to better mimic in vivo environments for kidney organoids is crucial for developing treatment strategies for ADPKD in the future.

Ongoing clinical studies have significantly contributed to the understanding of PKD2 function. The studies “A Possible Founding PKD2 Mutation Associated with Variable Phenotypes of ADPKD in Bergamo Province” and “Clinical and Molecular Description of PKD1 and PKD2 Mutation Negative Carriers in ADPKD” offer valuable insights into the phenotypic variability and genetic complexity of ADPKD, while also identifying new therapeutic and diagnostic targets. Other clinical trials, such as the “Study of Tamibarotene in Patients With ADPKD”, “A Study of RGLS8429 in Patients with Autosomal Dominant Polycystic Kidney Disease” and “Study of Empagliflozin in Patients with Autosomal Dominant Polycystic Kidney Disease (EMPA-PKD)”, provide significant scientific insights for the development of therapeutic agents for ADPKD. By evaluating the safety and efficacy of these candidate drugs, these studies hold the potential to advance therapeutic innovations in the field, ultimately offering more effective treatment options for patients with ADPKD.

**Table 1:** Summary of PKD2 roles in organ development.

Different Function of PKD2	Associated Proteins	Post Translational Modifications	Brief Mechanism	Reference
Left–right asymmetry	Bicc1, Ccr4, Dand5, Nodal, Lefty2, Pitx2	N/A	PKD2 regulates calcium signaling in the left-side cells of the LRO, leading to the degradation of Dand5 mRNA and the activation of nodal signaling, which ultimately establishes left–right asymmetry in internal organs.	[45,46,47,48,49,50]
Regulation of intracellular calcium homeostasis	Ryanodine receptors	Phosphorylation of Ser812 by Casein Kinase II	PKD2 on cilia responds to mechanical signals, leading to calcium release from the endoplasmic reticulum via Ryanodine receptors.	[74]
kidneys				
Regulation of cell proliferation in kidneys	Adenylyl cyclase 5/6, CREB, HEXIM1, cAMP, PKA, VEGF, ERK	Phosphorylation of Ser158 of HEXIM1	Decreased intracellular calcium in Pkd2 mutant kidneys reduces calcium-mediated inhibition of adenylyl cyclase, increasing cAMP levels, enhancing PKA activity, and stimulating cell proliferation.	[77,78,79,80,81,82,83,84,85]
Interaction with transcription factors	ID2, E2F, Rb	Phosphorylation of PKD2 (S812)	Phosphorylated PKD2 interacts with ID2, preventing it from inhibiting p21 activation, leading to reduced CDK2 activity and enhanced cell proliferation through E2F transcription factors.	[86,87,88]
Regulation of energy metabolism	Mitofusin 2 (MFN2)	N/A	PKD2 regulates mitochondrial function and calcium transfer efficiency, enhancing respiration and promoting cell proliferation. Reduced MFN2 expression can restore mitochondrial calcium transfer.	[89]
Maintenance of cell polarity	β-catenin, Axin2, c-Myc, E-cadherin	N/A	In Pkd2 mutant *mice*, increased expression of β-catenin, Axin2, and c-Myc indicates disrupted canonical Wnt signaling. This disruption leads to elevated cytoplasmic β-catenin levels, which impede renal epithelial polarization due to loss of E-cadherin-mediated cell–cell contacts.	[105]
Maintenance of renal structure in *zebrafish*	Scribble, GSK-3β, YAP	Phosphorylation at the N-terminal site of Pkd2	Pkd2 is located at the basal lateral membrane of the pronephric duct in *zebrafish*. Knockdown of Pkd2 reduces Scribble levels, inhibiting the Hippo signaling pathway and causing YAP to translocate to the nucleus, promoting glomerular cyst formation. Mislocalization affects structural integrity and function.	[99]
Cardiovascular system				
Regulation of valve formation in *zebrafish*	Camk2g, Klf2a	N/A	In *zebrafish*, early blood flow in the heart generates mechanical signals that are transduced by Pkd2 in atrioventricular endocardial cells. This regulates intracellular calcium ion concentration, activating Camk2g, which promotes Klf2a expression and valve formation.	[38,106,107]
Regulation of cardiac calcium signaling	RyR2, cardiac troponin I, phospholamban	N/A	In *mouse* cardiomyocytes, PKD2 interacts with RyR2, inhibiting its channel activity in the presence of calcium. This leads to decreased calcium concentration in the sarcoplasmic reticulum, enhancing PKA-mediated phosphorylation of cardiac troponin I and reducing phospholamban dephosphorylation, which alters myocardial contractility.	[180]
Cardiac protection during stress	Chromogranin B (CGB), ANP, BNP	Not specified	In stressed *mice*, PKD2 levels increase, correlating with elevated left ventricular mass and natriuretic peptides (ANP and BNP). PKD2-deficient *mice* fail to upregulate protective atrial natriuretic peptide, leading to persistent elevated left ventricular mass after stress cessation.	[112]
Regulation of vascular pressure through sensing mechanical forces	Calcium, calmodulin, protein kinase B, protein kinase C, α1-adrenoceptors	SUMOylation	PKD2, located in cilia of vascular endothelial cells, senses fluid shear stress, activating a pathway that includes calcium influx and nitric oxide production, leading to vascular dilation and reduced blood pressure. In arterial myocytes, PKD2 channels are activated by intravascular pressure, inducing sodium currents that result in vasoconstriction. SUMO-PKD2 localization to the cell membrane is regulated by intravascular pressure, which stabilizes vascular pressure through controlling PKD2 abundance. In heterozygous Pkd2 *mice*, oxidative stress impairs relaxation function.	[121,124]
Regulation of body axis development	sec23A, sec23B, sec24C, sec24D, Pde1a	N/A	Loss of Pkd2 function in *zebrafish* leads to dorsal curvature of the body axis, potentially due to excessive type II collagen accumulation in notochord sheath cells. PKD2 maintains calcium ion homeostasis and modulates collagen gene expression through the phosphoinositide 3-kinase signaling pathway. Overexpression of Pde1a reduces cAMP levels, mitigating axial curvature.	[154,155]
